# Editorial: Online and Offline Modulators of Motor Learning

**DOI:** 10.3389/fnhum.2017.00069

**Published:** 2017-02-21

**Authors:** Shahabeddin Vahdat, Genevieve Albouy, Bradley King, Ovidiu Lungu, Julien Doyon

**Affiliations:** ^1^Functional Neuroimaging Unit, University of MontrealMontreal, QB, Canada; ^2^KU Leuven, Movement Control and Neuroplasticity Research GroupLeuven, Belgium

**Keywords:** motor learning, memory, consolidation, sleep, brain stimulation, rehabilitation

What are the multitude of factors and processes that shape the acquisition and stabilization of a new motor skill? This is an important question that needs to be meticulously considered in order to design efficient paradigms for sports training programs as well as new rehabilitative protocols for restoring motor function following trauma or disease. Although the motor learning literature is abundant with research investigating the behavioral and neuronal determinants of online and offline motor learning (i.e., occurring during and after motor practice, respectively), an integrated view of the various factors influencing these determinants is not available in the literature. The aim of this Research Topic is therefore to address this gap and to bring together a set of articles that document how different factors modulate online and offline motor learning.

Critically, this special issue presents a wide range of modulators targeting both online and offline motor learning processes that can potentially be translated into clinical applications. Specifically, interventions including brain stimulation (Savic and Meier), exercise (Taubert et al.), the manipulation of the nature of motor practice (actual vs. imagination, Di Rienzo et al.), the timing of motor practice (de Beukelaar et al.), the training schedule (Müssgens and Ullén), the nature of the learned material (Du et al.), the cognitive load (Borragan et al.), the psychosocial context (Zemankova et al.), the availablity of visual (Rjosk et al.), or sensory (van Polanen and Davare) feedback all represent promising modulators of online motor learning processes. This special issue also reports interventions directly targeting offline processes, including the manipulation of post-practice vigilance and activity states, with the introduction of post-training sleep (Csabi et al.; Di Rienzo et al.; Malangre and Blischke) and exercise (Taubert et al.), but also the manipulation of the number and timing of the practice sessions after initial practice (triggering reactivation and reconsolidation processes, de Beukelaar et al.). Last, what makes this special issue unique is not only the variety of motor learning tasks investigated (from finer e.g., de Beukelaar et al. to grosser e.g., Malangre and Blischke), but also the diversity of populations studied [from children (e.g., Julius and Adi-Japha) to elderly (e.g., Zemankova et al.); in healthy but also pathological conditions (e.g., Csabi et al.; Zemankova et al.)].

With respect to the variety of tasks investigated, we would like to highlight two papers in particular that examined motor tasks that are highly relevant in clinical settings. Specifically, Rjosk et al. investigated whether mirror visual feedback can modulate a ball rotation task performed with the dominant or non-dominant hand; critically, these results have important implications in neurorehabilitation. Likewise, Malangré and Blischke investigated whether sleep facilitates the consolidation of gross motor sequence learning with a task in which subjects were required to fit a small peg into different target-holes. Consistent with previous literature using more laboratory-specific sequence learning tasks, they demonstrated offline performance improvements, which only occurred after an off-line period including sleep, but not wake.

From a lifespan perspective, two studies demonstrated that modulation of motor learning via developmental factors. Julius and Adi-Japha studied children in different age ranges and showed that successful motor learning depends on encapsulation of an initial, relatively accurate motor performance that could be improved throughout training. Csábi et al. investigated the effect of sleep-disordered breathing (SDB) on memory consolidation in children. Children with SDB exhibited intact motor sequence memory consolidation following sleep compared to healthy children, in contrast to previous reports in sleep-disordered adults.

As an example of intervention with important translational value, Savic and Meier reviewed current evidence supporting the modulatory effects of transcranial direct current stimulation (tDCS) on implicit motor learning. They outlined different parameters and mechanisms for tDCS to attain improved motor learning and consolidation. Likewise, Taubert et al. delineated the role of exercise, or more specifically endurance training, in optimizing motor learning. They provide a mechanistic link between exercise and motor learning-induced neuroplasticity at the systems, cellular, and molecular levels of brain organization. Furthermore, Di Rienzo et al. proposed in their review on the effects of motor imagery practice on motor learning that, as an execution-free training protocol can be a useful tool especially for the rehabilitation of patients with severe motor impairments.

Manipulation of the timing and schedule of motor practice, as well as of the nature of the learned information can also be introduced in clinical practice in order to modulate motor learning. Specifically, time spent awake before learning a new motor sequence task is believed to modulate motor learning. Using transcranial magnetic stimulation, De Beukelaar et al. measured changes in the excitability of the primary motor cortex following sequence learning either in the morning or in the evening. Their results support the view that during the day synaptic weights become saturated, which decreases the capacity for learning-induced changes in synaptic strength, as measured by corticomotor excitability. Using a similar motor task, Müssgens and Ullén investigated the effects of constant vs. variable training schedule on the extent of skill transfer to different conditions. Their results indicated that variable training led to greater general transfer to a new sequence of movements, likely attributed to more interference associated with constant training. Du et al. showed that learning a motor task governed by probabilistic rules is mainly driven by offline, rather than online, improvements in performance. This study highlights the importance of offline learning processes in more natural environments, where the desired sequence of movements depends on several unaccounted parameters.

Non-motor neural processes can also exert a substantial influence on motor learning processes and a subset of papers in this review have provided critical insights into this issue (Rjosk et al.; van Polanen and Davare). It has been proposed that cognitive control and motor learning compete for limited brain resources. In order to test this hypothesis, Borragán et al. instructed subjects to perform working-memory updating tasks with either high or low cognitive load before motor sequence learning. Their results indicated that high cognitive fatigue enhanced motor sequence learning. Zemankova et al. reviewed various psychosocial factors that might influence motor learning, and hence motor rehabilitation interventions, in Parkinson's disease. They elaborated on the contributions of social interaction, mindset and self-regulatory mechanisms and emotions on the effectiveness of motor training in more ecological environments, and highlighted their interactions with Parkinson's disease.

Modulation of offline processes supporting motor learning is also an interesting avenue in the quest of the optimization of motor behavior. Sleep has been shown to play an essential role in this offline process as it facilitates memory consolidation and stabilization of sequential motor skills. Several papers in this issue have documented the characteristics of sleep-dependent motor memory consolidation in relation to developmental factors (Csábi et al.), motor imagery (Di Rienzo et al.), and in gross motor tasks (Malangré and Blischke). Moreover, the study by de Beukelaar et al. showed that that reconsolidation of motor sequence memories, taking place post-training after a short re-exposure to the task, depends on both additional motor practice as well as the passage of time. Longer time delays, as opposed to a short delay, between re-exposure (reactivation) and interference practice were unable to de-stabilize the consolidated motor sequence memory trace. Hence, their findings support a time-dependent offline process in motor memory reconsolidation.

We believe that this special issue will increase our understanding of the physiological processes underlying motor learning and offers a more comprehensive view of how these processes can be modulated at different time points during the online and the offline learning periods. The integrated approach offered by this Research Topic may serve as a stepping-stone from which optimized neurorehabilitative approaches can be developed to help recovery of motor functions following injuries or neurodegenerative processes.

## Author contributions

SV, GA, BK, OL, and JD wrote the editorial article.

### Conflict of interest statement

The authors declare that the research was conducted in the absence of any commercial or financial relationships that could be construed as a potential conflict of interest.

